# MULTIFOCAL BULLOUS FIXED DRUG ERUPTION DUE TO FLUCONAZOLE

**DOI:** 10.4103/0019-5154.43212

**Published:** 2008

**Authors:** Amiya Kumar Nath, Balaji Adityan, Devinder Mohan Thappa

**Affiliations:** *From the Department of Dermatology and STD, Jawaharlal Institute of Postgraduate Medical Education and Research (JIPMER), Pondicherry - 605 006, India. E-mail: dmthappa@satyam.net.in/dmthappa@gmail.com*

Fixed drug eruption (FDE) due to fluconazole is extremely rare. We recently had a 35-year-old female patient who presented with multifocal bullous FDE due to fluconazole hitherto not reported in the literature.

**Fig. 1:**
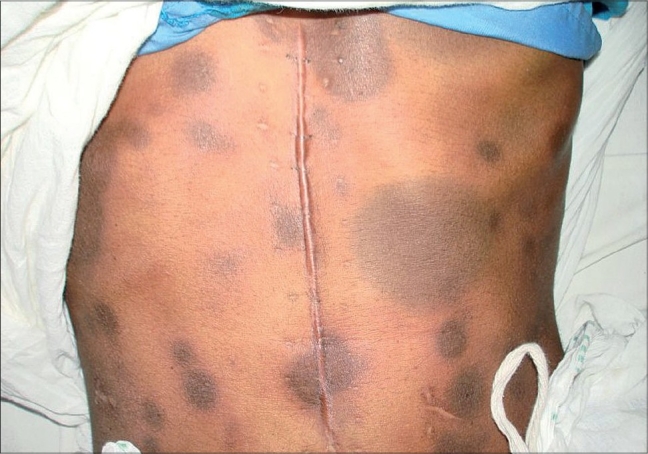
Multifocal fixed drug eruption due to fluconazole

A 35-year-old female, a known case of carcinoma stomach, operated and initiated on post-operative chemotherapy with cisplatin and adriamycin, presented with multiple itchy hyperpigmented elevated oval patches over the face, neck, trunk, upper limbs and lower limbs of three days duration following some medications for sore throat problem (details of the medication were not available at that time). On physical examination, she had multiple (200 in number) asymmetrically distributed, rounded to oval, hyperpigmented, edematous lesions of variable sizes surrounded by a rim of erythema over the body. Many lesions were bullous. She had a similar episode six months ago but at that time had developed only three lesions on the face and neck following oral medication for mouth problem. A diagnosis of multifocal bullous FDE was made. The skin lesions subsided over next one week leaving behind hyperpigmented macules without any intervention. Patch testing with cisplatin and adriamycin was negative. Oral rechallenge test to paracetamol and diclofenac sodium was negative. Subsequently, she was diagnosed to have oral candidiasis in our hospital and was prescribed oral fluconazole 200 mg on day one followed by 50 mg daily for two weeks. Within half an hour of intake of first dose, she developed itching in the skin lesions and in a couple of hours, developed reactivation of FDE lesions (Fig. 1). She was managed with systemic corticosteroids to control the reaction. Thus, a diagnosis of multifocal bullous FDE due to fluconazole was established.

Fluconazole is a triazole antifungal. It is fungisatic and is effective against many yeasts and dermatophytes.^1^ It acts by inhibiting 14 α-demethylase, a microsomal cytochrome P450 enzyme, in the fungal cell membrane.^1^ Despite it's widespread use, side-effects due to fluconazole are rarely reported. Side-effects are common when used in high doses (400 mg/day for months), especially in HIV individuals.^2^ Reported side-effects are nausea, vomiting, abdominal pain, diarrhea, elevated liver enzymes, mild to fatal hepatic necrosis, thrombocytopenia, agranulocytosis, leucopenia, eosinophilia, headache, dizziness, seizures, paresthesia, anaphylaxis, acute adrenal insufficiency, hypokalemia, teratogenicity, menstrual irregularities, and various cutaneous side effects like pruritus, exanthems, erythema multiforme, Stevens-Johnson syndrome, toxic epidermal necrolysis, exfoliative dermatitis, fixed drug eruption, angioedema, oral ulcers, purpura, and reversible alopecia.^2^

Most of the cutaneous side-effects due to fluconazole exist in isolated or a limited number of case reports. Fixed drug eruption due to fluconazole is extremely rare. To the best of our knowledge, there are only eight earlier reports of fixed drug eruption due to fluconazole in the literature.^3^–^5^

FDE is characterized by sudden onset of round and/or oval edematous, dusky-red, macules/plaques on the skin and/or mucosa arising precisely over the area of an earlier reaction due to the same drug.^6^ However, with subsequent exposure to the culprit drug, a few to numerous new lesions may appear and sometimes may become generalized. Acute lesions usually develop 30 min to eight hours after re-administration of the incriminating drug.^6^ After the initial acute phase lasting days to weeks, residual grayish or slate-colored hyperpigmentation develops.^6^ Various morphological types of lesions that may occur are morbiliform, scarlatiniform, erythema multiforme-like, Stevens-Johnson syndrome-like, eczematous, urticarial, nodular, vesicular, bullous (may become toxic epidermal necrolysis-like in severe cases), non-pigmenting and diffuse hypomelanosis.^6^ Confirmation of diagnosis requires re-challenge by topical provocation (in the form of patch test) and/or oral provocation test, of which oral provocation test is considered superior but unsafe.^6^

Our literature search on FDE due to fluconazole revealed that all cases reported so far had limited cutaneous or mucosal lesions. In contrast, our patient had extensive generalized lesions with many bullous lesions, a unique presentation of fixed drug eruption due to fluconazole which has not been described in the literature so far. With widespread use of any drug, new adverse effects come to light and hitherto unknown adverse effect may become more common occurrence. We recommend that fluconazole be included in the list of drugs causing multifocal bullous FDE so that similar cases may be diagnosed more often.
